# FONO SENSE: a technological resource for recording the auditory N400 component

**DOI:** 10.3389/fpsyg.2025.1565222

**Published:** 2025-06-24

**Authors:** Ana Luiza de Faria Luiz, Yara Bagali Alcântara, Isabela Tiezi Rombola, Simone Aparecida Capellini, Ana Claudia Figueiredo Frizzo

**Affiliations:** ^1^Objective Hearing Assessment Laboratory (LAAUD), Department of Speech and Hearing Sciences, School of Philosophy and Sciences (UNESP), São Paulo State University (UNESP), São Paulo, Brazil; ^2^Investigation Learning Disabilities Laboratory (LIDA), Department of Speech and Hearing Sciences, School of Philosophy and Sciences, São Paulo State University (UNESP), São Paulo, Brazil

**Keywords:** dyslexia, event-related potentials, N400, auditory evoked potentials, phonological processing, language development, electrophysiological assessment

## Abstract

This study aimed to develop a tool for phonological auditory electrophysiological assessment, focusing on the N400 component of Event-Related Potentials in adults and children with and without dyslexia. The cross-sectional analytical research, approved by the ethics committee (protocol n° 4.565.753), included 25 participants divided into three groups: 10 children with dyslexia (EG), 5 children without dyslexia (CG-s), and 10 adults without learning disorders (CG-a). The study was conducted in two phases. The first phase involved developing a mobile application with Congruent and Incongruent phonological tasks using words and non-words to assess reading, letter-sound relationships, syntactic-semantic integration, and lexical memory. In the second phase, participants performed auditory-linguistic tasks with acoustic stimuli (/ba/and/da/), combined with the app tasks, while the N400 potential was recorded using the Biologic’s Evoked Potential System (EP) with binaural stimulation in an oddball paradigm. The results showed a significant difference in latency between EG and CG-a for the incongruent task, with EG displaying delayed latency. Only CG-a exhibited a significant latency reduction in the incongruent task. No significant differences in amplitude were observed between groups or factors. In conclusion, the application effectively elicited the N400 potential in all groups, demonstrating shorter latencies in adults compared to children, both with and without dyslexia.

## 1 Introduction

Auditory information processing is linked to the way the brain receives, organizes and interprets sound information. Individuals with dyslexia may have difficulty with auditory processing, especially in the ability to discriminate and manipulate sounds. Difficulties in these skills interfere with the way speech sounds and phonological awareness are understood (Barreiro and Momensohn-Santos, 2010; Pereira and Cavadas, 1998). Therefore, the processes of decoding and encoding auditory stimuli are predictors of the development of written language and, if there are deficits in these processes, the chance of having school difficulties during the literacy process is high (American Speech-Language-Hearing Association, 2005).

The International Dyslexia Association (2002) defines dyslexia as a specific learning disability that is neurobiological in origin. It is characterized by difficulties with accurate and/or fluent word recognition and by poor spelling and decoding abilities. These difficulties typically result from a deficit in the phonological component of language, often unexpected in relation to other cognitive abilities and the provision of effective classroom instruction. Secondary consequences may include problems in reading comprehension and reduced reading experience that can impede growth of vocabulary and background knowledge.

Different theories that seek to explain the etiology, one of which is due to a change in auditory processing (Evans, 2006). Children with dyslexia have difficulties due to a deficit in the processing of speech sounds, which affects their ability to discriminate, remember and perceive auditory sounds. These aspects are directly related to auditory processing. Auditory discrimination involves categorizing sounds based on their similarities and differences. Auditory memory is involved in the storage and retrieval of sound information. Auditory perception involves the reception and interpretation of combined sounds, including the perception of words (Dally, 2006; Kujala et al., 2006; Olivares-García et al., 2005).

To objectively assess the processing of auditory and linguistic information by the auditory nervous system, the measurement of auditory evoked potentials has been used. Late Evoked Potentials are responses of electrical activities that occur in response to an acoustic stimulus and the execution of a task and are directly linked to higher cortical functions such as the brain’s attention to sound, auditory discrimination, immediate memory, and decision-making and reasoning intrinsic to cognition (Kraus and McGee, 1999; Hall, 2006). The late and endogenous components of the late AEP, such as N2, P3, and N400, reflect the influence of higher cognitive and cortical processes on auditory processing. These endogenous components reveal the action or contribution of top-down processes, that is, guided by contextual information and expectations and decision-making, and are therefore called Event-Related Potentials (ERPs) (McPherson, 1996; Patel and Azzam, 2005; Martin et al., 2007).

The N400 ERP is a negative deflection, after the presentation of the linguistic account that begins 200–300 milliseconds (ms) after a word has been presented auditorily or visually and peaks after approximately 400 ms. Although the N400 response is often associated with semantic anomaly, it can be elicited by most meaningful stimuli, including single words, nonwords, faces and pictures, and its amplitude can vary according to the incongruence between the stimulus and the context (Lau et al., 2008).

First described in Kutas and Hillyard (1980) during an incongruent reading task associated with a linguistic context, it is currently known that the N400 component acts minimally on two aspects: semantic and phonological integration and access to information from long-term memory (Kutas and Federmeier, 2000). So, the N400 is used to investigate how the brain processes and understands language and can provide insights into the organization and dynamics of semantic representation in the human brain.

In Brazil, there is a technological restriction for eliciting the N400, especially in clinical Auditory Evoked Potentials equipment. Based on this demand, an application (APP) denominated FONOSENSE was developed to generate the component through linguistic tasks and complex stimulation paradigms. The technology presented here is a tool for the Speech Therapy/Audiology clinic that can expand the evaluation proposals and the field of Neuropsychology in Brazil. The hypothesis of the study is that the new task proposed in the FONOSENSE APP, which presents sounds, words and non-words, in the visual and auditory modalities, would be capable of generating the N400 component.

In addition to the technological innovation itself, the APP platform is independent and compatible with all clinical equipment for recording auditory evoked potentials, which opens up a range of application possibilities, and is a facilitating method for the field of research. The incorporation of technologies, especially mobile applications, in speech therapy and neuropsychology has proven to be fundamental for improving assessments and interventions in speech therapy clinics and related areas. These tools offer innovative approaches that enhance the effectiveness of health and education procedures used in patients with communication disorders.

Electrophysiology also benefits from these technological innovations, allowing more detailed assessments of auditory and cognitive functions and the monitoring of therapeutic interventions. The association of electrophysiological measures using speech sounds and the possible future association with neuroimaging makes it possible to provide insights into the brain mechanisms underlying auditory and language disorders and a deeper understanding of the correlations between behavior and neurophysiological phenomena, enriching diagnosis and therapeutic planning (Regaçone et al., 2013).

Thus, this article proposes Fono Sense as a new technological tool to generate and evaluate the N400 component of the ERP and presents a pilot study in adult readers and in children who read and children who have difficulty reading.

## 2 Materials and methods

The study was carried out after approval by the Research Ethics Committee of the São Paulo State University “Júlio de Mesquita Filho” (UNESP), Marília, São Paulo, Brazil. CAAE numbers 43381620.8.00005406 and 4,565,753. Application registered as a Computer Program at the National Institute of Industrial Property (INPI) under process no. BR512022000382-1. This is a cross-sectional analytical pilot study. 25 participants were subdivided into three groups: (I) Experimental Group (EG): 10 students with an interdisciplinary diagnosis of phonological subtype Dyslexia; (II) Adult Control Group (CG-a): 10 adults without dyslexia, without any learning disorders and/or other comorbidities; (III) Students Control Group (CG-s): five students without dyslexia, without any learning disorders and/or other comorbidities.

The participants in this study are children with an interdisciplinary diagnosis of developmental dyslexia. All children were diagnosed by a team of Investigation Learning Disabilities Laboratory – LIDA/FFC/UNESP, composed of at least three professionals: speech therapist, neuropsychologist and neurologist or child psychiatrist.

The diagnosis followed DSM-5 criteria (American Psychiatric Association, 2013) confirmed through individually administered standardized performance measures and comprehensive clinical assessment with manifestation of persistent difficulties in oral reading fluency, reading comprehension, written expression and spelling; impaired academic skills and below expectations for the student’s chronological age; normal levels of intellectual functioning [Intelligence Quotient greater than approximately 90 (±5 points margin of measurement error)] and no response to intervention (RTI Model).

The exclusion criteria to rule out individuals were: presence of middle ear abnormality detected by tympanometry; not classified as type A, to ensure that sound transmission occurred without deficit in the peripheral auditory system; and/or word recognition score below 92% (Jerger et al., 1968; Jerger, 1970); occurrence of other associated comorbidities and other problems of development evidenced by the anamnesis. Both CG-a and CG-s could not have dyslexia, other learning disabilities or other physical or intellectual comorbidities.

### 2.1 Development of *FONOSENSE*

The application created and registered in 2020 is compatible with the Android system for mobile platforms, the Flutter language, and used on a 7-inch computer during the research experiment. The interface was developed for use on conventional auditory evoked potential equipment to support speech-language pathologists and professionals from other areas such as neuropsychology, who are interested in brain functions and information processing. The proposed technological resource is used to evaluate aspects related to sound perception during an auditory-phonological task, which involves attention and discrimination of sounds followed by a forced choice in a semantic context.

**Figure 1:**
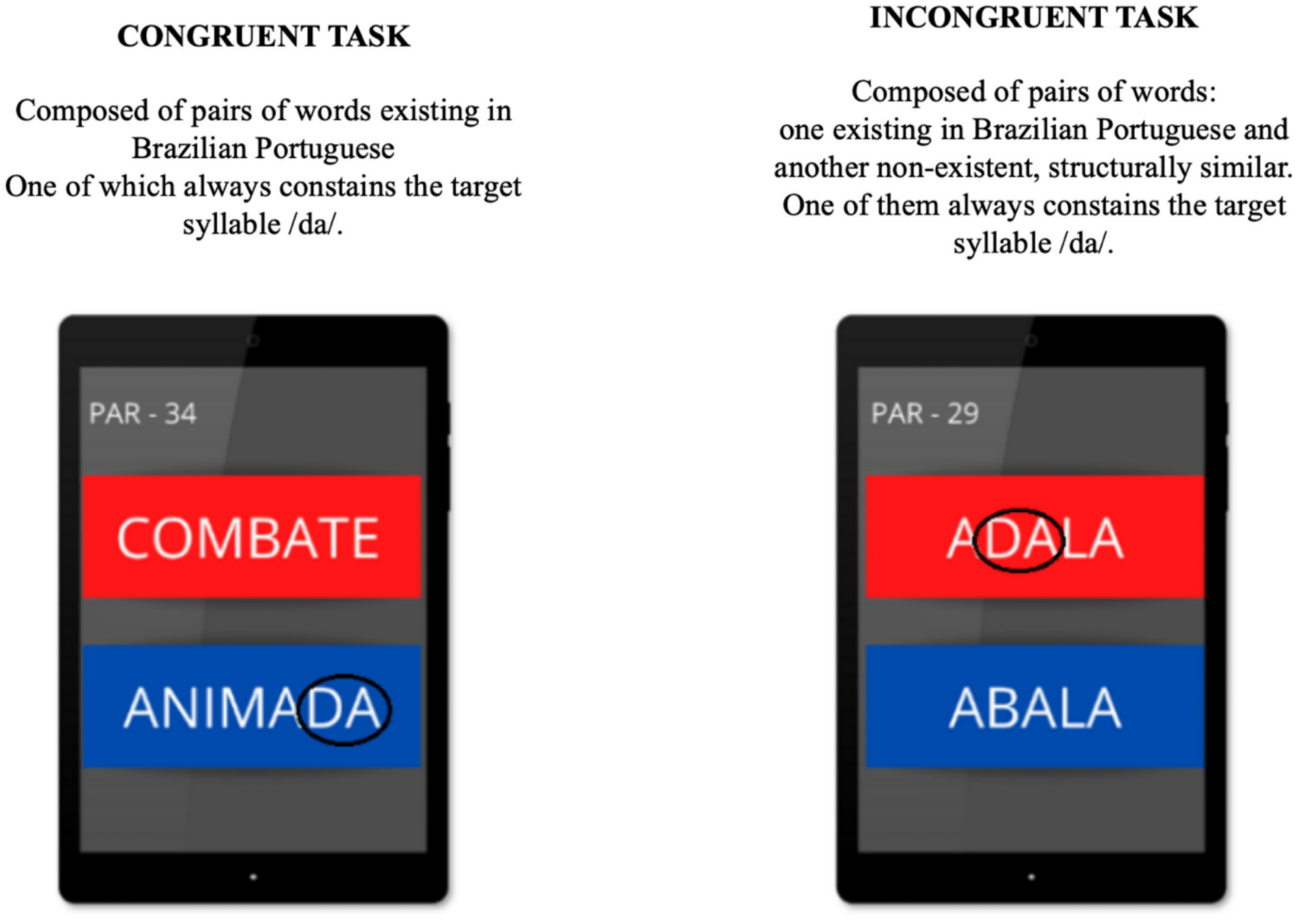
Example of the forced choice task of words and non-words. Syllable /da/ of the words circled in black. Source: FONO SENSE APP, 2021.

The application (APP) has a total of 100 pairs of selected words, which were divided equally into 50 pairs of only words (congruent task) and 50 pairs of words and pseudowords (incongruent task). Care was taken to ensure phonetic balance, using monosyllabic, bisyllabic and trisyllabic words and pseudowords, in the CV, CVCV and CVCVCV structures, which were used as visual stimuli in the APP. During the test, the PEA software presented random sounds, target syllables /da/ and non-target syllables/ba/as stimuli, and recorded the count of stimulus presentations. The sequence of stimuli was synchronous with the visual stimuli, words and non-words in the APP. At the time of the presentation of the sounds, immediately after generation, the volunteer performed the forced choice and decision-making by selecting the corresponding word or non-word containing the syllable/da/, touching the screen, which changed with each choice/touch. In the end, the total count of the 50 sound stimuli presented corresponded exactly to the 50 visual stimuli (syllables) selected for each type of task, congruent (words) and incongruent (non-words) (see Figure 1).

### 2.2 Registration of event-related potentials

Event-related auditory evoked response recordings were performed using a two-channel auditory evoked potential (AER) device, Biologic’s Evoked Potential System (EP) (Seattle, WA/USA). Other materials such as copper electrodes, abrasive paste and conductive paste were also used.

The electrodes were assembled according to the International Standard 10–20 (Jasper, 1958), with the active electrode positioned at Cz (central), reference electrodes A1 and A2, respectively left and right ear and Ground electrode, positioned at Fpz (forehead) with impedance lower than 5 k ohms. The recording of Event-Related Potentials ERP was done in a room with acoustic and electrical protection. The simulation was done with speech sounds presented in insert headphones (ERA-2A) at an intensity of 70 dB HLPe, syllables in phonological contrast [ba] (75%) x [da] (25%) randomized, with stimulation rate of 0.9 stimuli per second and a total of 200 stimuli and a total of 50 [da] target stimuli. The ERP recording was made in a 1,000 ms window, with 1-30 Hz filtering with a rejection level of 30 μV, as the volunteer was in an alert state.

Natural speech stimuli were recorded at 48 kHz in Praat® (version 4.2.31) (Boersma and Weenink, 2003) in an acoustically treated room of the Linguistics Laboratory and saved in .wav format compatible with the Evoked Potential System - Biologic Navigator® for auditory stimulation and response recording. The total duration of the syllables [ba] and [da] was 180 ms.

The positive and negative peaks of the EEG wave were recorded in a time window that ranged from −100 to 1,000 ms. Then, the previous components, the complex of exogenous components P1-N1-P2 and the late N2, P3, were identified and marked, which guided the subsequent identification of the N400 around ms according to pre-established criteria (McPherson, 1996; Kutas and Federmeier, 2000).

Qualitative variables are described by relative frequency distribution (%) and 95% confidence interval (95%CI). The relationship between qualitative variables was analyzed by Fisher’s exact test. Quantitative variables are described by mean and 95%CI. Homogeneity of variances was analyzed by Levene’s test. To compare the mean between groups, a one-way ANOVA was performed followed by the Bonferroni post-hoc test. To analyze the effect of group and factor (congruent and incongruent), as well as the interaction (group versus factor), a mixed repeated measures ANOVA was performed based on the assumption of homogeneity of covariance matrices by the Box test followed by Bonferroni post-hoc comparisons. The effect size for ANOVA was determined by Eta (Pereira and Cavadas, 1998). The Eta (Pereira and Cavadas, 1998) values were interpreted as: 0.10 small; 0.25 medium; and 0.40 large (Espirito Santo and Daniel, 2018). The significance level adopted was 5% and the data were analyzed using SPSS software (version 24.0).

## 3 Results

Table 1 describes the profile of the sample of research participants who underwent the PRE assessment and the measurement of the N400 neural component.

**Table 1:** Sample profile description.

Variable	Category	EG (*n* = 10)	CG -s (*n* = 5)	CG -a (*n* = 10)
		*F*	*F*	*F*
Age	Younger Students (9 years to 10 years and 11 months)	10	5	0
	Older Students (20 years to 24 years and 2 months)	0	0	10
Gender	Feminine	5	3	5
	Masculine	5	2	5
	Complete fundamental	0	0	0
Scholarship	Incomplete fundamental	10	5	0
	Complete higher education	0	0	10
	Incomplete higher education	0	0	0

EG, Experimental Group; CG-a, Control group - adults; CG-s, Control Group - Students; F, frequency.

## 4 Data analysis

**Figure 2:**
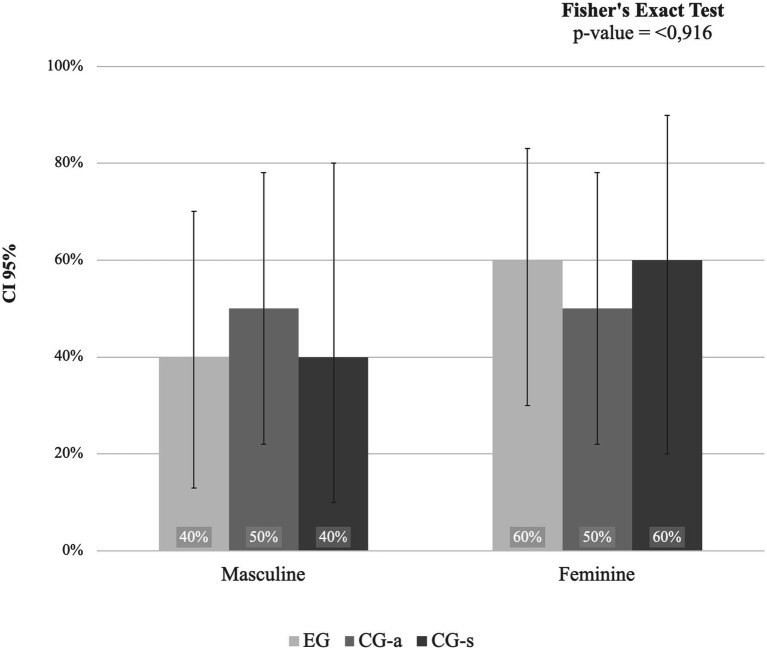
Analysis of the association of the relative frequency distribution (%) and 95% confidence interval (95%CI) of sex within the group. EG, Experimental Group; CG-a, Control Group - adults, GC-s, Control Group – students. Source: prepared by the author.

Figure 2 shows the analysis of the association of the relative frequency distribution (%) and 95% confidence interval (95%CI) of sex within the group. The *p*-value was calculated using Fisher’s exact test for association.

There was no significant association between sex and group, that is, the groups have similar sex ratio distributions.

**Figure 3:**
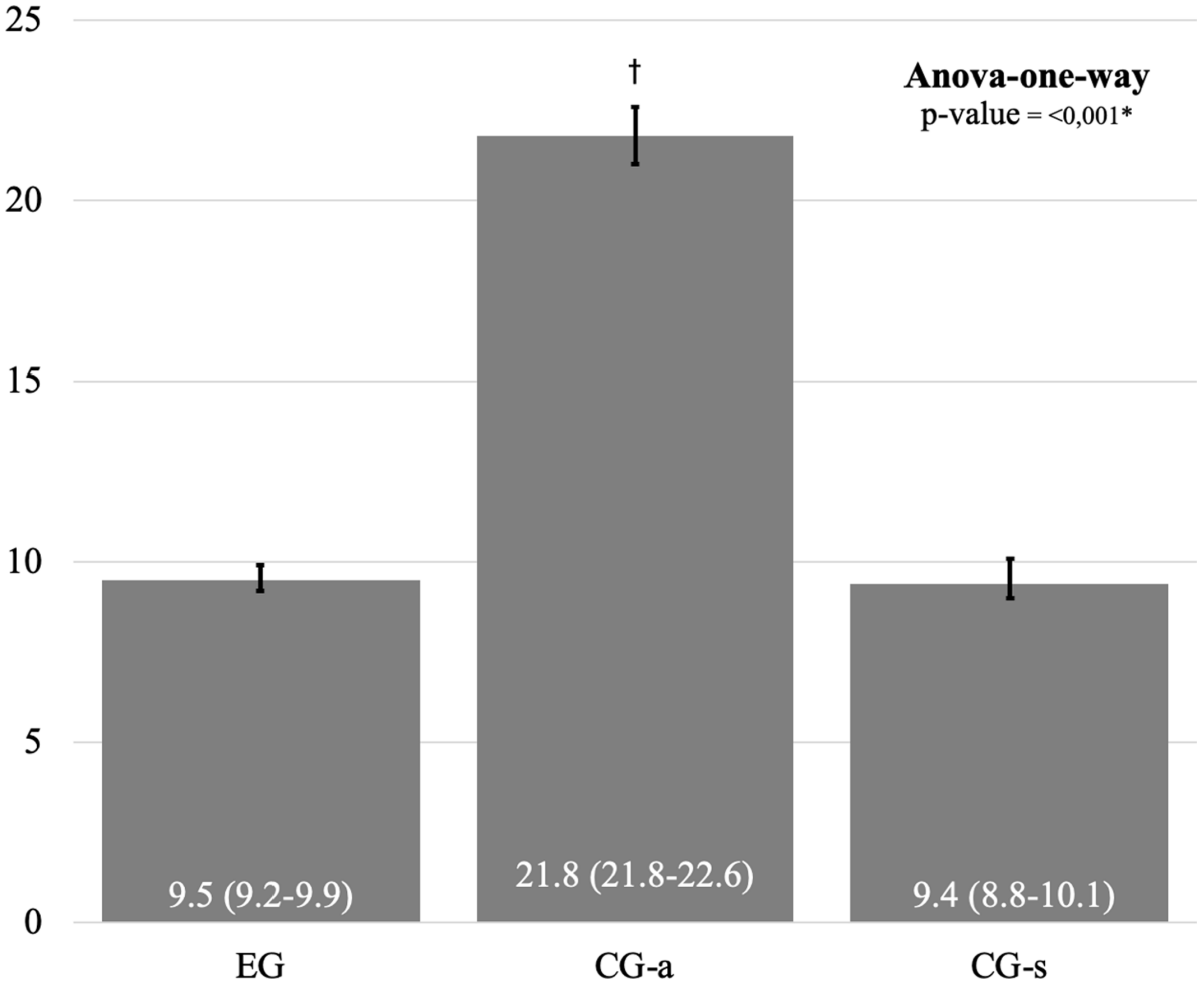
Comparison of mean and 95% confidence interval (95%CI) for age between groups. Legend: EG: Experimental Group; CG-a: Control Group - adults, GC-s: Control Group - students; *, significant difference between groups by one-way Anova test; †, significant difference in relation to the other groups by Post-hoc and Bonferroni test. Source: prepared by the author.

Figure 3 below shows the comparison of the mean and 95% confidence interval (95%CI) for age (years) between the groups.

Table 2 presents the comparison of the mean and 95% confidence interval (95%CI) for the latency of the N400 component between the groups and by type of task. The results indicate significant differences between the groups (*p* = 0.005) and a significant interaction between group and type of task (*p* = 0.0039). A shorter N400 latency value was observed for the adult group.

**Table 2:** Comparison of mean and 95% confidence interval (95%CI) for N400 component latency between groups and by task type.

Variable	Group			Factor		Anova		
				Congruent (words)	Incongruent (words/ no words)	Group	Factor	Interaction
						*p*-value	*p*-value	*p*-value
N400 LT	EG	Mean		491.55	506.17a	0.005**	0.291	0.039*
		CI 95%	UB	468.77	495.21			
			LB	514.34	517.13			
	CG-a†	Mean		474.32	453.23b			
		CI 95%	UB	455.81	430.94			
			LB	492.83	475.54			
	CG-s	Mean		491.79	477.64			
		CI 95%	UB	467.52	446.87			
			LB	516.06	508.42			
	Total	Mean		484.71	479.29			
		CI 95%	UL	473.25	465.36			
			LB	496.16	493.23			

LI lower limit for 95%CI; LS upper limit for **indicates significant difference between groups regardless of task type by repeated-measures mixed ANOVA for *p*-value ≤ 0.05. * Indicates significant interaction between group and task type by repeated-measures mixed ANOVA for *p*-value ≤ 0.05. Different letters indicate significant differences between groups by Bonferroni post-hoc test for *p*-value ≤ 0.05. † Indicates significant difference between factors within the group by Bonferroni post-hoc test for *p*-value ≤ 0.05. LT, latency; EG, Experimental Group; CG-a, Control Group- adults; CG-s, Control Group - students; CI, confidence interval; UB, under bound of confidence interval calculator 95%; LB, lower bound of confidence interval calculator 95%.

Table 3 shows the comparison of the mean and 95% confidence interval (95%CI) for the amplitude of the N400 component between the groups and by type of task. Regarding the amplitude, there was no significant difference between the groups and congruence and incongruence factors for the CG-a, CG-s and EG groups.

**Table 3:** Comparison of the mean and 95% confidence interval (95%CI) for the amplitude of the N400 neural component between groups and by type of task.

Variable	Group			Factor		Anova		
				Congruent (words)	Incongruent (words/ no words)	Group	Factor	Interaction
						*p*-value	*p*-value	*p*-value
N400 LT	EG	Mean		−3.47	−4.52	0.308	0.232	0.926
		CI 95%	UB	−5.65	−7.16			
			LB	−1.28	−1.87			
	CG-a†	Mean		−2.33	−3.69			
		CI 95%	UB	−3.09	−5.03			
			LB	−1.57	−2.35			
	CG-s	Mean		−3.73	−4.27			
		CI 95%	UB	−4.85	−6.19			
			LB	−2.61	−2.36			
	Total	Mean		−3.07	−4.14			
		CI 95%	UB	−3.94	−5.23			
			LB	−2.19	−3.05			

LT, latency; UB, under bound of confidence interval calculator 95%; LB, lower bound of confidence interval calculator 95%; EG, Experimental Group; CG-a, Control Group - adults; CG-s, Control Group - students; CI, confidence interval. † indicates a significant difference between factors within the group by the Bonferroni post-hoc test for *p*-value ≤ 0.05.

**Figure 4:**
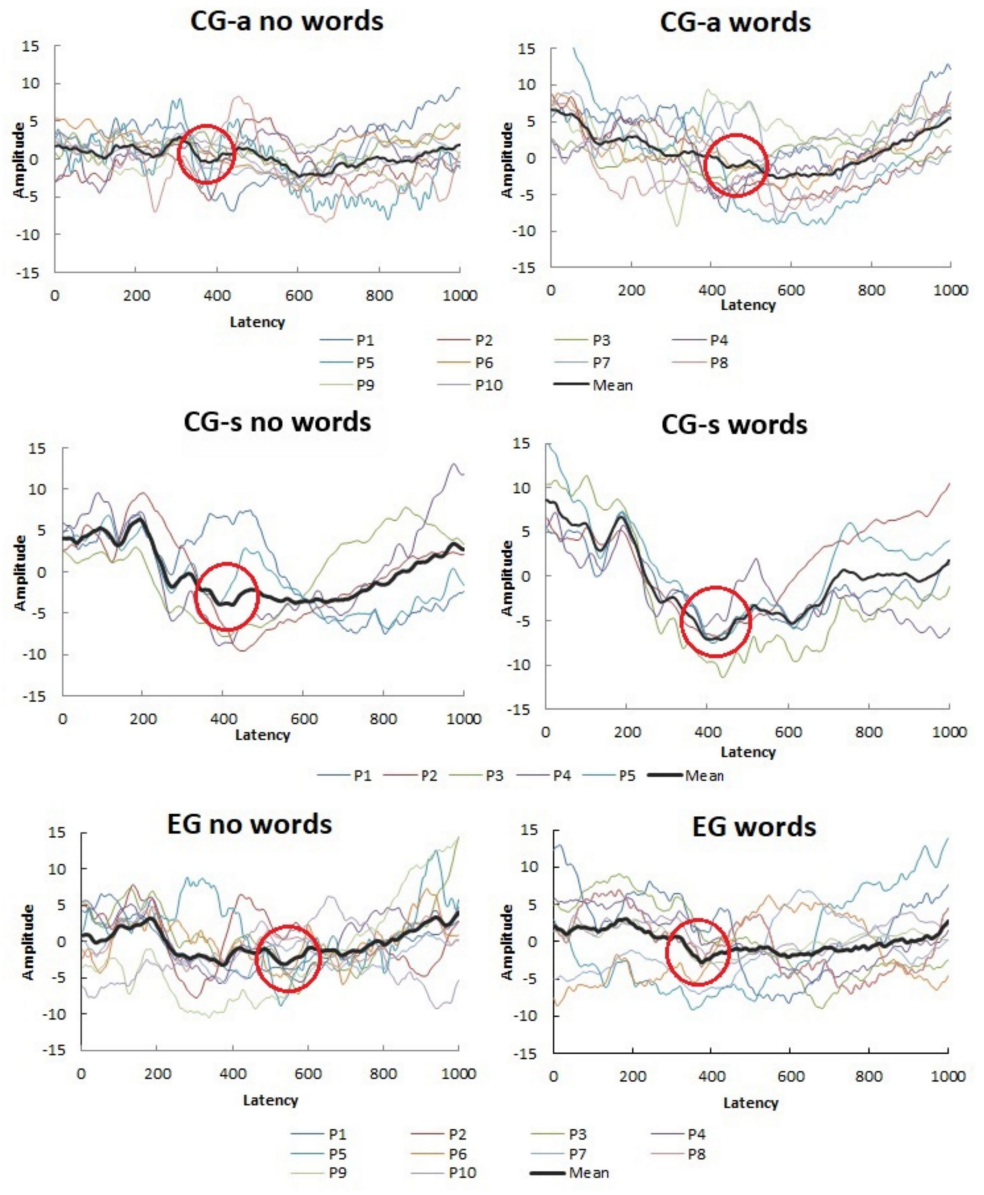
Grand average of PRE-N400 exam records for the three groups. CG-a, control group adults; CG-S, control group Students; EG, experimental group. Source: prepared by the author.

Figure 4 shows the ERP recording, highlighting the presence of the N400 component across the three research groups: EG, CG-s and CG-a, for both congruent and incongruent tasks.

## 5 Discussion

The application of the FONOSENSE APP enabled the observation of the N400 component, a typical negative peak of the ERP with a latency of around 400 ms. In the pilot study, this response was observed in both tasks in the groups of adults, dyslexic children and non-dyslexic children participating. On the other hand, the comparative analyses of the study revealed significant differences for types of task and groups.

The N400 latency measurement relative to the speed of information processing in milliseconds of adults showed significantly shorter latency for the incongruent task compared to children. In parallel in the comparison of interaction between groups, there was a significant difference in relation to the incongruent task of the group of children with dyslexia in relation to the group of adults. There was also a significant increase in latency in the group of dyslexic children for the incongruent task in relation to the group of adults.

These findings are consistent with faster processing speed and more efficient linguistic processing in adults, especially in the incongruent task with more complex linguistic demands, while the differences in latency and amplitude suggest slower phonological-semantic integration and/or compensatory mechanisms for task performance in dyslexic children (França, 2005).

When comparing the amplitude aspect relative to the magnitude of electrical activity involved in performing the task, in this study, no significant differences were observed between the N400 measurements in the congruent and incongruent tasks between the groups or in the interaction between them. A study (Lau et al., 2008) showed that in relation to amplitude, when there is a reduction, it is reflecting greater ease in accessing memory and lexical information. In this study, the mean N400 amplitude values were less negative for the group of dyslexic children in relation to children without dyslexia. These findings confirm the difficulty dyslexic children have when faced with the demand for linguistic incongruence due to their neurological alteration that affects the phonological aspect (Maess et al., 2006).

We hypothesize that distinct patterns in N400 morphology emerge across groups, influenced by their respective reading development status. Children who are in the early stages of reading acquisition rely predominantly on the phonological route, which leads to a more robust N400 with greater amplitude. This reflects the increased cognitive load involved in phonological decoding and semantic integration. In contrast, adults, who have a fully developed reading system and predominantly engage the lexical route, do not require the same level of decision-making effort, resulting in a less negative and lower amplitude N400. For dyslexic children, differences in N400 latency and amplitude suggest slower phonological-semantic integration and/or compensatory mechanisms during task performance. Although amplitude differences were not statistically significant, a trend was observed, indicating that, despite processing difficulties, there is sustained effort to efficiently integrate information.

The PRE is useful for the auditory-linguistic functional assessment of various populations, from those with typical development to children and adults with language and learning disorders. Dyslexia is one of the most well-known learning disorders, characterized by a marked neurologically-based difficulty in reading and writing during development. One theory regarding the etiology of dyslexia suggests that the disorder stems from an alteration in information processing, which explain the impaired perception of speech sounds and, consequently, deficits in phonological awareness that underlie reading difficulties. The literature presents neuroimaging studies that show a different pattern of brain activation in these individuals when compared to individuals without dyslexia in tasks of semantic congruence and incongruence of sentences, findings that are also confirmed by this study (Martin et al., 2016).

The N400 is the ERP capable of assisting in the investigation of phonological alterations in language disorders, since it is sensitive to phonological variables and auditory performances in recognizing presented words. Therefore, the characteristics of the N400 could be used as indicators of the time course of activation of semantic and phonological codes and, consequently, of the effectiveness of therapeutic methods (Praamstra and Stegeman, 1993).

Regarding the type of task, it was observed that the N400 is even more evident in the incongruent task for both adults and children with and without dyslexia, since this component reflects the measurement of the endogenous potential related to the semantic and phonological processing of a linguistic auditory task (with semantic incongruence) and occurs based on an initial process of discrimination and attention to the distinct acoustic characteristics of the linguistic stimulus characterized by the P1-N1-P2 complex, followed by a cognitive process dependent on the complexity of the task (França, 2005).

Finally, the results of the pilot study showed that adults have a greater ability to distinguish more complex linguistic tasks than children due to the maturation of the auditory pathways and auditory cortex, the already consolidated and efficient reading acquisition process, and the extensive language experience as a facilitator of flexical access that influences the N400 amplitude (Lau et al., 2008). As for dyslexia, electroencephalographic investigations have already shown changes in the peak of the N400 PRE of incongruent sentences compatible with impairment and slowness in semantic linguistic processing (França, 2005).

The use of electroencephalographic recording techniques and equipment is essential to assess functional aspects related to information processing at cortical levels. ERPs are recorded by capturing bioelectrical signals from the human nervous system, which requires the use of sophisticated technological resources and systems. Evoked potential systems perform a complex process of stimulation, acquisition, digitization, measurement and summation of biological electrical activity. The use of external devices coupled to the equipment, which generate complex stimuli with speech signals, allows a specific assessment of the quality of linguistic and semantic processing.

In speech therapy, the use of applications facilitates the assessment of language and auditory processing, providing unified and interactive protocols that make the process more dynamic and accurate. One example is the FonoLingo application, developed by an interdisciplinary team, which assists in the assessment of speech and language, unifying protocols and promoting greater interactivity with the patient (Ferreira et al., 2019).

The elicitation and analysis of late neural components, involving a cognitive task that generates a new “event” in the brain. This event is directly related to the perception of sound and the execution of the task, effectively characterizing the ERP. In the specific case of the N400, the negative deflection that follows the P1-N1-P2 complex and the P300 requires a linguistic task that involves phonological, syntactic and semantic aspects for its peak to be evidenced.

The event-related potential (ERP) was initially described in language processing studies and revealed that modulations of the N400 component are more easily evoked when there is a mismatch between a stimulus, known as syntactic-semantic mismatches in accordance with the findings of this study. It is widely accepted that this mismatch, responsible for the N400 effect, reflects processing based on sensory and meaning association (Kutas and Hillyard, 1980).

In this context, Horowitz-Kraus and Breznitz (2008) investigated the electrophysiological activity associated with reading in individuals with dyslexia and proficient readers and identified, as in this study, a late N400 in incorrect responses in both groups of dyslexic readers and suggest a distinct electrophysiological pattern in reading tasks in this population.

Corroborating this perspective, Robichon et al. (2002) analyzed the electrophysiological pattern associated with the difficulty of adults with dyslexia in integrating the meaning of words within sentences, through the recording of the N400. Their results showed that dyslexic participants presented greater amplitude and latency of the N400 in the parieto-central region in response to incongruent sentences, as well as to congruent and incongruent words, in the latency range between 350 and 500 ms. This increase in the latency of the N400 was interpreted as a compensatory mechanism necessary to integrate words within the context of the sentence, reinforcing the idea that individuals with dyslexia present differentiated language processing.

It is noteworthy that the present that the present study was conducted with a non-probabilistic convenience sample, composed of all available participants who met the inclusion criteria, totaling 25 sample elements. Considering this sample size, a pilot approach was proposed. To verify the adequacy of the sample, a sample size calculation was performed a posteriori based on the observed effect size (0.256 for LT and 0.007 for AT), using the repeated measures ANOVA test. Considering a type I error margin and a power of 80%, it was estimated that an ideal sample size would be 42 participants for LT and 49,000 for AL. For future studies, a minimum of 42 participants is recommended for LT. However, for AT, the required sample size is high and unfeasible, making it essential to conduct new pilot studies for better sample sizing.

Regarding the use of one-way ANOVA or repeated measures ANOVA, although the groups are unequal and the sample is small, which may increase the chance of type I and type II errors, the homogeneity assumptions were met and support the use of ANOVAs. Thus, the use of nonparametric tests or correction of variance homogeneities would not bring better performance in reducing type I and II errors.

Nevertheless, the results achieved provide a basis to support the use of this application in Speech-Language Pathology, contributing to the advancement of scientific knowledge in this area and the expansion of clinical health resources with application in educational demands and thus expanding the proposals for neuropsychological assessment and strengthening the understanding of this theme in Brazil. The clinical application of FONOSENSE is promising, as it offers the opportunity to improve the accuracy and reliability of the assessment procedures performed by speech-language pathologists and audiologists and the offer of new instruments in the field of Neuropsychology in Brazil.

Based on this evidence, this instrument may play a crucial role in the diagnosis and monitoring of language disorders, providing a more objective and promising approach. In the future, the incorporation of FONOSENSE into clinical practices may represent a valuable innovation, as it enables the monitoring of the therapeutic progress of individuals with dyslexia and the evaluation of the effectiveness of the treatments used.

These findings also raise new questions and highlight the need for further studies, which aim to expand the sample and apply the technology developed in FONO SENSE in other clinical contexts and validation studies, including usability aspects such as ease of navigation, clarity of the user interface, and responsiveness across different devices. Additionally, the study seeks to explore the application’s use in interventions in individuals with dyslexia, in order to monitor therapeutic progress and evaluate treatment effectiveness.

Thus, FONOSENSE emerges as a notable contribution to the field of speech-language pathology, offering substantial benefits to professionals and patients. Expanding its use to other clinical contexts, in addition to expanding the sample studied, represents a promising path for the continued development of this application and its application in different populations, further driving scientific advancement and the improvement of therapeutic care.

## 6 Conclusion

The application of the FONO SENSE application allowed the observation of the N400 component, a typical negative ERP peak with a latency of around 400 ms. In the pilot study, this response was observed in the groups of adults, dyslexic children and non-dyslexic children participating in the pilot study. Furthermore, the analyses revealed distinct responses in both the latency and amplitude of the evoked potentials among the groups studied, particularly between adult readers and children with dyslexia. The latter exhibited differences in the N400 component during the incongruent task, consistent with impairments and delays in semantic linguistic processing. Although a positive response was observed, conducting the study with a larger sample is essential to ensure greater reliability of the findings and enhance their clinical applicability.

## Data Availability

The raw data supporting the conclusions of this article will be made available by the authors, without undue reservation.
